# Imidazolium-based ionic liquids as dispersants to improve the stability of asphaltene in Egyptian heavy crude oil

**DOI:** 10.1038/s41598-023-44237-w

**Published:** 2023-10-11

**Authors:** Alaa Ghanem, Maher I. Nessim, N. A. Khalil, Raghda A. El-Nagar

**Affiliations:** 1 PVT Lab, Production Department, Egyptian Petroleum Research Institute, Nasr City, 11727 Cairo Egypt; 2 Egyptian Petroleum Research Institute, PVT Services Center, Nasr City, 11727 Cairo Egypt; 3 Petroleum Testing Lab, Analysis & Evaluation Department, Egyptian Petroleum Research Institute, Nasr City, 11727 Cairo Egypt

**Keywords:** Green chemistry, Chemical synthesis, Energy, Crude oil, Chemistry, Energy science and technology

## Abstract

Deposition of asphaltene aggregates can easily depress the oil production, because it may clog the wellbores, annulus, pipelines, and surface facilities. Moreover, asphaltene molecules have a negative effect on the catalytic reactions in the refinery process. Therefore, in this work, three different ionic liquids (IL-H, IL-CH_3_, and IL-NO_2_) were synthesized, and characterized using FT-IR and NMR spectroscopy to evaluate their efficiency as asphaltene dispersants. The thermal gravimetric analysis of the prepared ILs showed that IL-H, IL-NO_2_, and IL-CH_3_ were thermally stable up to 280 °C. The ILs showed good dispersion activity of the petroleum asphaltenes, where the asphaltene onset precipitation (AOP) was changed from 7.5 to 10.5, 11, and 13.5 ml added n-heptane after the use of IL-H, IL-NO_2_, and IL-CH_3_, respectively. Moreover, the colloidal instability index of crude oil was changed from 0.92 (unstable asphaltene) to 0.69 (stable asphaltene). It is noted during the experiments that the presence of an alkyl chain attached to the ionic liquid moiety increases the efficiency of the dispersant. This may be owing to the formation of π–π^*^ with asphaltene molecules due to the presence of electron donating group. Quantum chemical parameters were calculated for the prepared ILs, and the theoretical data confirmed the experimental results.

## Introduction

Asphaltene is a complex mixture of high molecular weight organic compounds that occurs naturally in crude oil and its properties vary significantly depending on the source of crude oil^1–3^. These compounds are heavy, dense, highly viscus substances with a large proportion of aromatic and hetero-aromatic rings, and they are characterized by their insolubility in n-alkanes and their solubility in aromatic solvents such as toluene and xylene^4^. It is well known that asphaltene is the heaviest and most complex fractions in crude oil, therefore numerous operational problems in the production, transportation, and refining of crude oil could be arises easily. These problems include deposition, fouling, precipitation, and emulsion stabilization^4–6^. Many factors promote asphaltene deposition such as the change in pressure, temperature, or compositional analysis of crude oil^7^. The change in crude oil pressure occurs naturally during depletion or production. While the change in temperature occurs when the asphaltene-containing crude oil comes in contact with a surface that is colder than the oil temperature. Different techniques of enhanced oil recovery could easily change the compositional analysis of the crude oil^8,9^. All these factors encourage the deposition of solid asphaltene particles, which can lead to blockages in pipelines, valves, and production equipment^10^.

The development and implementation of a profound chemical injection strategy to mitigate asphaltene-related issues are paramount for a successful and sustainable fluid production if routine operating conditions are within the asphaltene instability range^11^. Because of the energy crises, the petroleum sector has shifted to exploiting the heavy and extra-heavy oil, despite the fact that there are other options such as syngas, natural gas, and solar energy^12–16^. Several studies have been carried out to investigate the problems caused by the accumulated asphaltenes and to come up with suitable remedies^17^. Different aromatic solvents such as toluene and xylene are being used to remediate deposition and remove unwanted asphaltene deposits to restore production^17^. Ethanol and methyl ester oleate are among the solvents used to remove asphaltene from the pore spaces^18^. One different and effective method to dissolve asphaltene or to inhibit asphaltene precipitation in crude oil is to use chemicals designed to mimic the activity of resins either by adsorbing on the asphaltene surface or interacting with asphaltene molecules via electrostatic attraction or hydrogen bonding^19^. Many researchers investigated the use of nanofluid based TiO2 and SiO2 as asphaltene inhibitors^20,21^. It was found that the 80% TiO2 nanocomposit can adsorb the asphaltene molecules on its surface, which delay the onset of asphaltene flocculation^21^.

Recently, there are many applications of the ionic liquids in the petroleum industry, especially in the treatment of asphaltene-related issues in crude oil production, transportation, and refining, and showed promising results^14,22–26^. In the last few decades, ionic liquids (ILs) granted a significant attention and have been considered the most efficient alternative to ordinary organic solvents. This is because of their unique and highly tunable physico-chemical properties, such as non-flammability, low vapor pressure, very good thermal stability, and high ionic conductivity^25,27^. Therefore, ionic liquids (ILs) are efficient solvents for inorganic and organic constituents especially asphaltenes, moreover ILs are good asphaltene inhibitors^28,29^. In addition, ionic liquids have the ability to disturb the asphaltene aggregates enabling the release of light hydrocarbons and improve the flow ability of the crude oil^30^. Ionic liquids are low melting salts, which consist of organic cations and organic or inorganic anions^31^. The organic cations comprise asymmetric N-cyclic compounds such as imidazolium and pyridinium derivatives, while anions include various simple inorganic anions such as halides, tetrafluoroborate, methansulfonate, hexafluorophosphate, and more complex organic ones^32^. These anions can be suitable for most temperature and pressure conditions, and they are available with a varying degree of hydrophobicity. In addition, it was reported that the asphaltene stability is directly proportional to the net negative charge of the anion^26^. ILs are increasingly being explored for particular advantages in pace, specificity, and yield rather than just as a substitute for organic solvents in a variety of solvent applications today^29^. Many researchers discussed the functionalization of ILs as asphaltene inhibitors and dispersants, where specific functional groups are introduced to the cations and anions^23,33–36^. El-Hefnawy et al.^37^ used hydrophobic ionic liquids based on the alkyl and aryl imidazolium cation and carboxylic asphaltene as anion as asphaltene inhibitors. They found that the aryl ionic liquid can act as solubilizing agent for the asphaltene molecules and has more efficiency than the aliphatic one. According to Liu et al.^38^, the most potent ILs as asphaltene dissolver should have high aromaticity containing both anion and cation with hydrogen bond acceptor. In 2005, Hu and Guo^39^ studied the effect of alkyl pyridinium-based ionic liquid to disperse asphaltene. They found that the ILs with low density of cation charge have a neglected effect on the asphaltenes, due to the cations with low density charge does not have the ability to form stable complexes with asphaltene molecules^39^. Later in 2009, Boukherissa et al.^40^ investigated the effect of acidic ionic liquids as asphaltene inhibitors and assumed that the dispersion power was due to the formation of hydrogen bonds and charge transfer complexes with asphaltene molecules. This approach allows ILs to be tailored to specific applications. Moreover, they discussed the economic and environmental advantages of ILs over traditional solvents, such as their recyclability, low volatility, and high stability. Mahtar et al.^41^ synthized ionic liquid baseds on the biopolymer (lignosulfonate) and evaluated them as asphaltene inhibitors. The addition of long alkyl chain to the ILs could increase their surface-active characteristics^35^, in this regard Ghanem et al., investigated the effect of alkylated ionic liquids based on imidazolium sulfonate as asphaltene dispersants. They found that increasing the alkyl chain, increasing the efficiency of the ILs, where long-chain IL molecules can self-assemble in an aqueous solution to form micelles due to their molecular composition^29^. In case of imidazole-based ILs, attaching at least one long chain, such as C_10_–C_16_, to the imidazolium ring produces surface-active molecules, which are referred to as ionic-liquid based surfactants, or ILBSs^28^. While according to Boukherissa et al., the optimum ionic liquid should have an alkyl chain with eight carbon atoms at least^40,42^.

In this study, a series of ionic liquids based on imidazole were synthesized: 2-ethyl-1-hexyl-3-(2-oxo-2-(p-tolyl) ethyl)-1H-imidazol-3-ium, 2-ethyl-1-hexyl-3-(2-(4-nitrophenyl)-2-oxoethyl)-1H-imidazol-3-ium, and 2-ethyl-1-hexyl-3-(2-oxo-2-phenylethyl)-1H-imidazol-3-ium, named IL-CH_3_, IL-NO_2_, and IL-H, respectively. To the best of our knowledge, this is the first time that donor–acceptor characteristics of the prepared ionic liquids have been modified by the addition of an electron-donating group (CH_3_) in IL-CH_3_ and an electron-withdrawing group (NO_2_) in IL-NO_2_ to examine their impact on the inhibition of asphaltene precipitation. The prepared ionic liquids were evaluated as dispersants of petroleum asphaltenes using viscosity measurements and by calculating the colloidal instability index of the crude oil system. Quantum chemical parameters such as energy gap, softness, and hardness were calculated to investigate the reactivity of the prepared ionic liquid. The obtained results were utilized to understand the effect of different functional groups of the ionic liquids on asphaltene aggregates.

## Experimental

### Materials and methods

Ethyl acetate (98%), Acetonitrile (HPLC grade), acetone (AR) (Alfa Aesar), Potassium hydroxide (Pellets) (98%), acetic acid glacial (99%), (PioChem), 2-Chloroacetophenone (98%), 2-ethyl-1*H*-imidazole (99%), 1-bromohexane (98%), 2-chloro-1-p-tolyl-ethanone (95%) (Merck), 2-Chloro-1-(4-nitrophenyl)ethanone (95%), filter paper (whattman, 42 mm). All reagents were utilized as received without any further purification. Asphaltenic crude oil sample was collected from the eastern dessert fields in Egypt and was used after twenty minutes of agitation for better homogeneity.

#### Preparation of 1-hexyl-2-methyl-1H-imidazole

1-hexyl-2-methyl-1H-imidazole was synthesized using the same technique as our prior work^43^. In this experiment, 50 ml of acetonitrile was used to dissolve a known portion of 2-ethylimidazole and KOH (pellets) while stirring at room temperature for 30 min. Then, with constant stirring, an equivalent amount of 1-bromhexane was added dropwise. After that, the mixture was continuously stirred for 3 h finally, it was filtered to remove any byproduct. The final product was subjected to vacuum vaporization.

#### Synthesis of ionic liquids (IL-H, IL-CH_3_, and IL-NO_2_)

**Figure 1:**
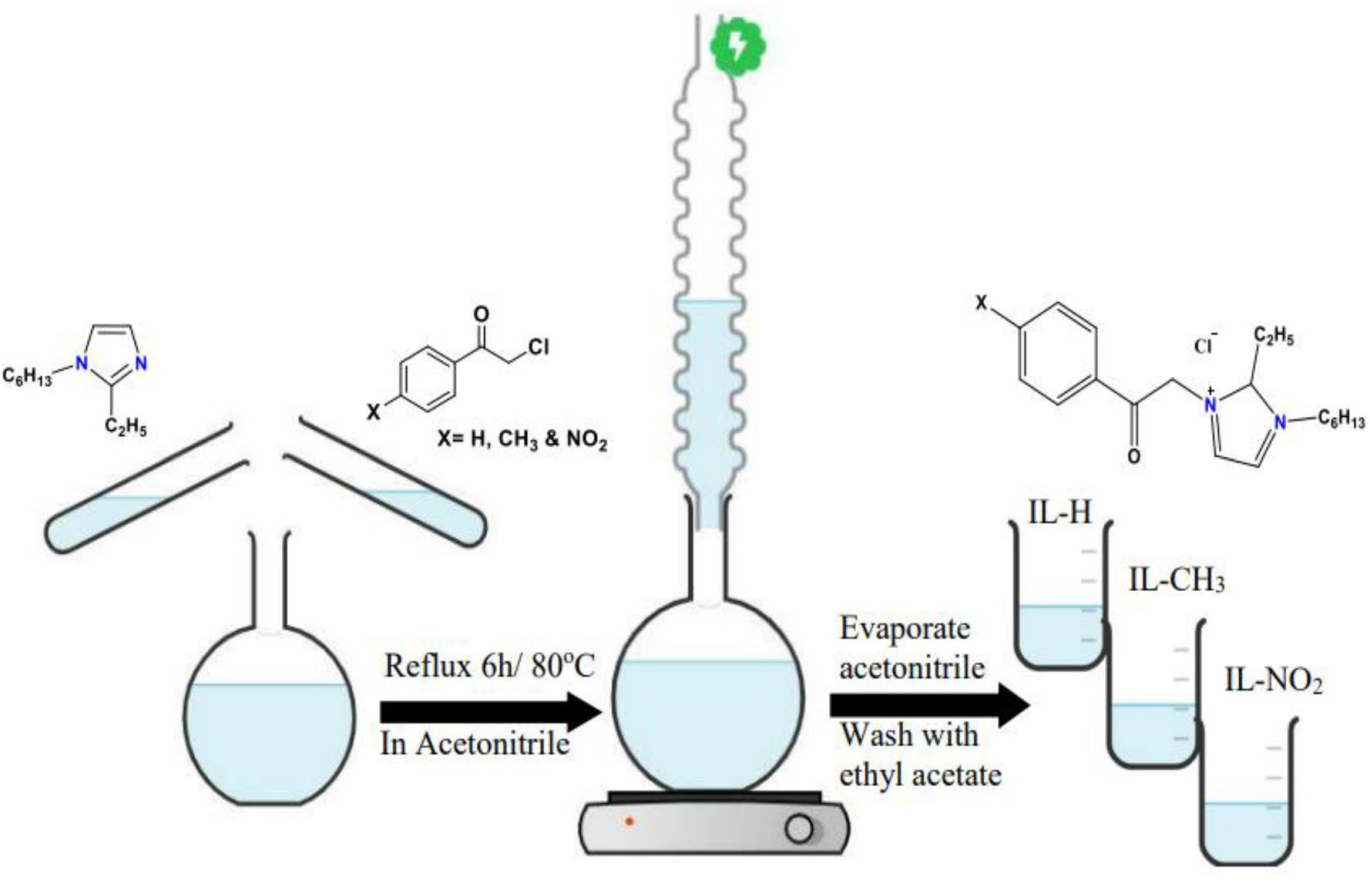
Synthesis of ionic liquids (IL-H, IL-CH_3_, and IL-NO_2_).

As shown in Fig. 1, three different ionic liquids (IL-H, IL-CH_3_, and IL-NO_2_) were formed by refluxing a solution of an equivalent amount of 1-hexyl-2-ethyl-1H-imidazole and 4-acetophenone derivatives in acetonitrile (50 ml) for 6 h at 80 °C. The acetonitrile was then evaporated and the obtained ILs were dried under vacuum after being washed many times with ethyl acetate. Table 1 displays the yield and the molecular structures of the produced ILs.

**Table 1:** Molecular structures and IUPAC names of the prepared materials.

IL	Yield, %	Molecular weight	Chemical name	Molecular structure
IL-H	81.5	335	2-Ethyl-1-hexyl-3-(2-oxo-2-phenylethyl)-1H-imidazol-3-ium	
IL-CH_3_	80.7	349	2-Ethyl-1-hexyl-3-(2-oxo-2-(p-tolyl) ethyl)-1H-imidazol-3-ium	
IL-NO_2_	81.9	380	2-Ethyl-1-hexyl-3-(2-(4-nitrophenyl)-2-oxoethyl)-1H-imidazol-3-ium	

### Characterization of the synthesized ILs

The chemical structures of the prepared ILs have been verified via spectroscopic instruments such as Nuclear Magnetic Resonance (^1^H-NMR, ^13^C-NMR, and HSQC-NMR), which were conducted using AVANCE-II NMR, 400 MHz, Bruker, Germany. In addition, FT-IR spectra of the ILs were recorded in the range of 4000–400 cm^−1^, using FT-IR spectrometer (Nicolet IS-10—Thermofisher, USA). Thermal Gravimetric Analysis was determined in the temperature range of 10–600 °C with a heating rate equal to 10 °C min^−1^ under nitrogen, via TGA-550 TA Instrument.

### Treating the asphaltenic crude oil by the prepared ionic liquids

**Figure 2:**
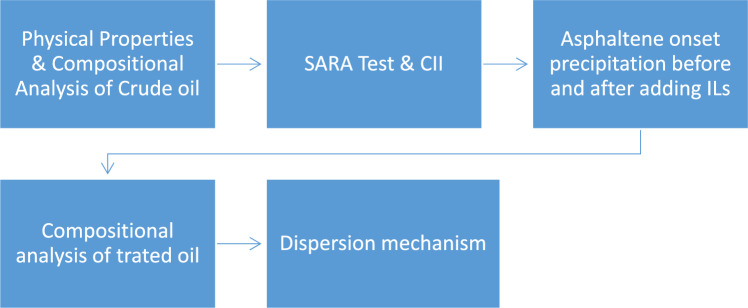
Workflow comprises the main steps to evaluate the ILs as asphaltene dispersants.

The prepared ionic liquids have been studied as dispersants for aggregated asphaltene to treat the crude oil according to the following workflow (Fig. 2).

### Crude oil characterization

Table 2 demonstrates the petrochemical properties of the crude oil sample. The data reveals that the crude oil sample is characterized by high viscosity and density, which worsen its flow properties, especially inside the pipelines.

**Table 2:** The petrochemical properties of the asphaltenic oil sample.

Experiment	Standard method	Result
Density at 15.56 °C API	ASTM D-4052	0.9476 17.68
Kinematic viscosity @ 40 °C, cSt	ASTM D-445	443.08
Molecular weight	…	361
Wax content, weight %	UOP/64	0. 95
Pour point temperature, °C	ASTM D/97	15
Sulphur content, %	ASTM D/4294	3.85

#### SARA test and colloidal instability index (CII)

SARA test (Saturates, Aromatic, Resin, and Asphaltenes) is used to analyse the main components of the crude oil sample, where asphaltene fraction content is determined according to IP-143, and SAR fractions are determined according to ASTM D2007^44^.

The obtained data from SARA test can be used to determine the colloidal instability index of the asphaltenic oil sample, which determine the instability index of asphaltene components in the crude oil sample. The colloidal instability index (CII) is the ratio between the summation of both fractions; asphaltene and saturates to the summation of both fractions; aromatic and resin as shown in Eq. (1)^45^. It is stated that the asphaltene is stable if the CII value is lower than 0.7, while it is unstable if the CII value is higher than 0.9. The values between 0.7 and 0.9 is considered as meta stable conditions.

1 $$Colloidal \;Instability \;Index \left( {CII} \right) = \frac{Asphaltenes\; Content + Saturates\; content}{{Resins \;Content + Aromatics \;Content}}$$

#### Compositional analysis of crude oil

The compositional analysis of the crude oil sample (up to C_40_) before and after treatment was conducted via Perkin Elmer-Clarus 500. The instrument is equipped with a flame ionization detector (FID) to detect the hydrocarbon components up to C_40_. The used column is a selective PIONA capillary column, which its length is 100 m and its internal diameter 0.25 mm.

### Asphaltene onset precipitation

Viscosity measurements was used to determine the asphaltene onset precipitation. The measurements were conducted using a stabinger viscometer (SVM 3001-Anton Paar), where a series of crude oil was titrated with an asphaltene precipitant (n-heptane) to cover the range of precipitant from zero to 100%. The instrument was firstly calibrated using a reference material. Each run was repeated three times and the average was calculated. The resultant values were plotted against the n-heptane concentration.

## Results and discussion

### Nuclear magnetic resonance for IL-H, IL-CH_3_ and IL-NO_2_

#### ^1^H-NMR spectra

**Figure 3:**
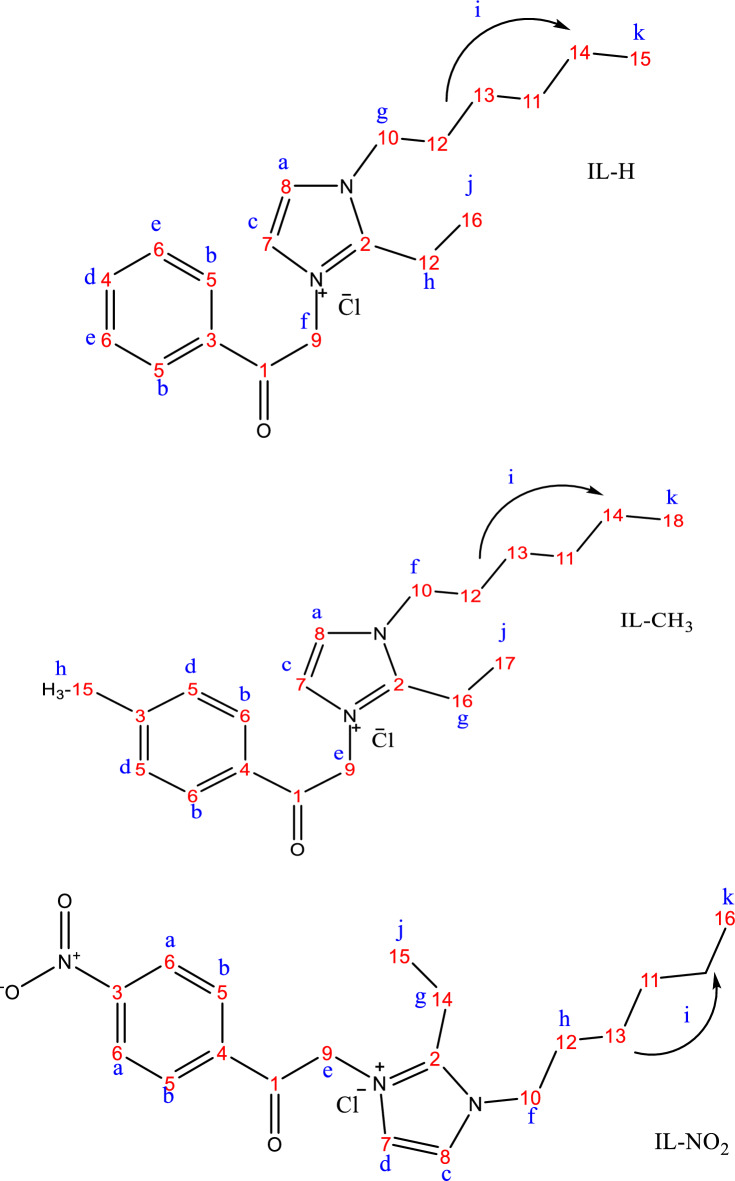
Protons and carbon atoms distributions in the prepared ILs Structures according to^1^H-NMR and ^13^C-NMR.

**Figure 4:**
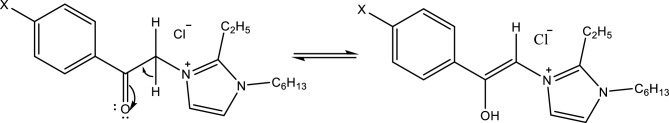
Keto-enol form structure of IL-H, IL-CH_3_, and IL-NO_2_, where X is H, CH_3_, and NO_2_, respectively.

**Figure 5:**
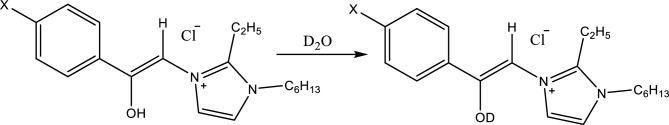
Deuterated structure of IL-H, IL-CH_3_, and IL-NO_2_, where X is H, CH_3_, and NO_2_, respectively.

Figure 3 shows the chemical structures of the synthesized ILs, including the distribution of protons and carbon atoms. While the chemical shift values were presented in Table 3. ^1^H-NMR data recorded in Table 3 confirmed the existence of the synthesized ILs as follows: the protons of O–H groups were detected at 14.05, 13.98, and 13.99 ppm for IL-H, IL-CH_3_, and IL-NO_2_, respectively. The de-shielded protons (a–d) that appeared at 8.07 and 7.91 ppm for IL-H and IL-CH_3_, respectively, related to the presence of the carbonyl group as electron withdrawing group, and increased to 8.33 ppm effected by the ortho-position of nitro group in the case of IL-NO_2_. The aliphatic protons of the alkyl chain possess the lowest δ values with singlet spin because of similarity. Methylene protons, beside the olefinic ones (f in IL-H, e in IL-CH_3_ and IL-NO_2_) were recorded at 5.34, 5.28, and 5.73 ppm (Table 3). The olefinic protons, which were detected at 6.19, 6.18, and 6.22 ppm, confirmed the presence of IL-H, IL-CH_3_, and IL-NO_2_ in keto-enol form (Fig. 4). The presence of Keto-enol form was also indicated by D_2_O-NMR. Deuterium (D) rapidly replaced the OH proton, and the OH was converted to OD, resulting in the disappearing of ^1^H proton as shown in Fig. 5.

**Table 3:** The chemical shifts (δ, ppm) of IL-H, IL-CH_3_ &IL-NO_2_.

Cpd	O–H	a	b	c	D	e	f	G	h	i	j	k
IL-H	14.05 (s)	8.07 (d)	7.98 (d)	7.71 (d)	7.65 (d)	7.53 (d)	5.34 (s)	4.93 (t)	2.58 (s)	1.90 (m)	1.20 (s)	0.82 (s)
IL-CH_3_	13.98 (s)	7.91 (d)	7.91 (d)	7.69 (d)	7.63 (d)	5.28 (s)	4.12 (t)	2.65 (s)	2.48 (s)	1.87 (m)	1.21 (s)	0.80 (s)
IL-NO_2_	13.99 (s)	8.33 (d)	8.30 (d)	7.94 (d)	7.72 (d)	5.73 (s)	4.14 (t)	2.67 (s)	1.71 (m)	1.24 (s)	0.82 (s)	–

where, s, d, m, and t represent the spin multiplicity.

#### ^13^C-NMR spectra

^13^C-NMR data confirmed the chemical structure of IL-H, IL-CH_3_ and IL-NO_2_. Table 4 showed the values of the chemical shifts in ppm related to ^13^C-NMR for scattering carbon atoms of the synthesized ILs.

**Table 4:** ^13^C NMR chemical shift values in ppm of carbon atoms in IL-H, IL-CH_3_ and IL-NO_2_.

	IL-H	IL-CH_3_	IL-NO_2_		IL-H	IL-CH_3_	IL-NO_2_
C1	191.1	186.6	185.6	C11	31.3	43.8	31.2
C2	145.2	144.2	143.6	C12	29.1	31.3	29
C3	134.7	143.4	143.4	C13	28.7	31	28.6
C4	134.3	129.9	136.2	C14	28.5	28.3	28.3
C5	128.8	129.5	135.9	C15	25.4	25.69	25.4
C6	128.4	129.3	130.6	C16	22.1	22.1	21.8
C7	120.9	128.1	121.9	C17	–	21.2	–
C8	116.9	122.2	118.1	C18	–	14	–
C9	54.33	118.1	46.8	COH	147.2	144.7	150.8
C10	47.4	46.8	43.3		65	56.3	56.3

#### Hetero-nuclear single-quantum correlation spectroscopy (HSQC)

**Figure 6:**
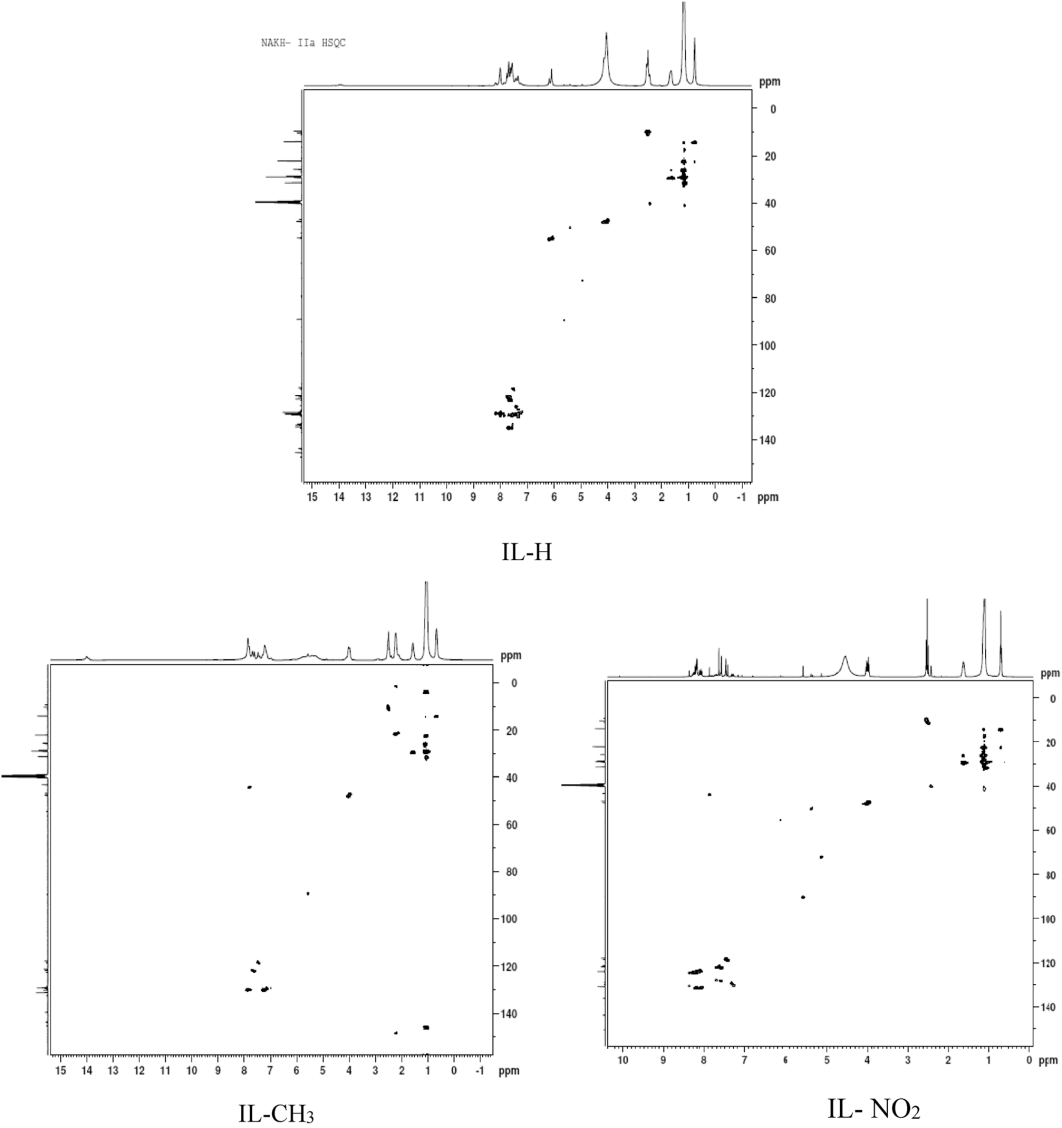
HSQC of IL-H, IL-CH_3_, and IL-NO_2_.

HSQC is a frequently two-dimensional (2D) spectroscopy technique represented by two axis for ^1^H and ^13^C used for organic molecule strictures confirmations^35^. The observed cross peaks indicate a single peak attributed to each definite proton that attached to the considered carbon atom. Figure 6 showed the 2D correlation for IL-H, IL-CH_3_, and IL-NO_2_.

### FT-IR spectra for IL-H, IL-CH_3_, and IL-NO_2_

FT-IR values of IL-H, IL-CH3, and IL-NO2 were reported in Table 5. The wide broad bands at 3389, 3389, and 3428 cm^−1^ related to OH from moister for IL-H, IL-CH_3_, and IL-NO_2_ respectively.

- The spectra of stretching vibrational C–N bands were detected at 1449, 1464, and 1463 cm^−1^. C–C vibrations were recorded at 1595, 1602 and 1601 cm^−1^ for IL-H, IL-CH_3_, and IL-NO_2_, respectively.
- The in-plane bands of imidazole rings (bending/vibrational) were noticed at 1230–1192 (For IL-H), 1272–1194 (For IL-CH_3_) and 1267–1200 cm^−1^ (For IL-NO_2_). While the bending vibrations bands of out of plane appeared at 757 (For IL-H), 751 (For IL-CH_3_) and 717 cm^−1^ (For IL-NO_2_).
- The vibrational distortion values of the imidazole rings were observed at 687, 698 and 671 cm^−1^ for IL-H, IL-CH_3_ and IL-NO_2_ respectively.

**Table 5:** FT-IR data of IL-H, IL-CH_3_, and IL-NO_2_.

Wave number, Cm^−1^														
Cpd	–OH	C-H aromatic stretching	C-H aliphatic stretching	C=O	C=C aromatic	C–NO_2_	C–C Aromatic	Imidazolium ring zone						
								C–C	C–N	C–H in plane bending	C–H out plane bending	Ring deformation in plane bending	Aromatic bending zone	Ring deformation out of plane
IL-H	3389	3062	2924 2854	1687	1532	–	659	1595	1449–1281	1230–1192	1159 757	995 922	830 790	686
IL-CH_3_	3389	3028	2922 2853	1685	1529	–	664	1602	1464–1319	1272–1194	1091 751	979 920	833 788	696
IL-NO_2_	3428	3066	2923 2853	1704	1524	1525	657	1601	1463–1321	1267–1200	1108 717	992 917	855 786	674

The absorption band at 1525 cm^−1^, which is attributed to C-NO_2,_ was appeared in case of IL-NO_2_ only.

### Thermal gravimetric analysis (TGA)

**Figure 7:**
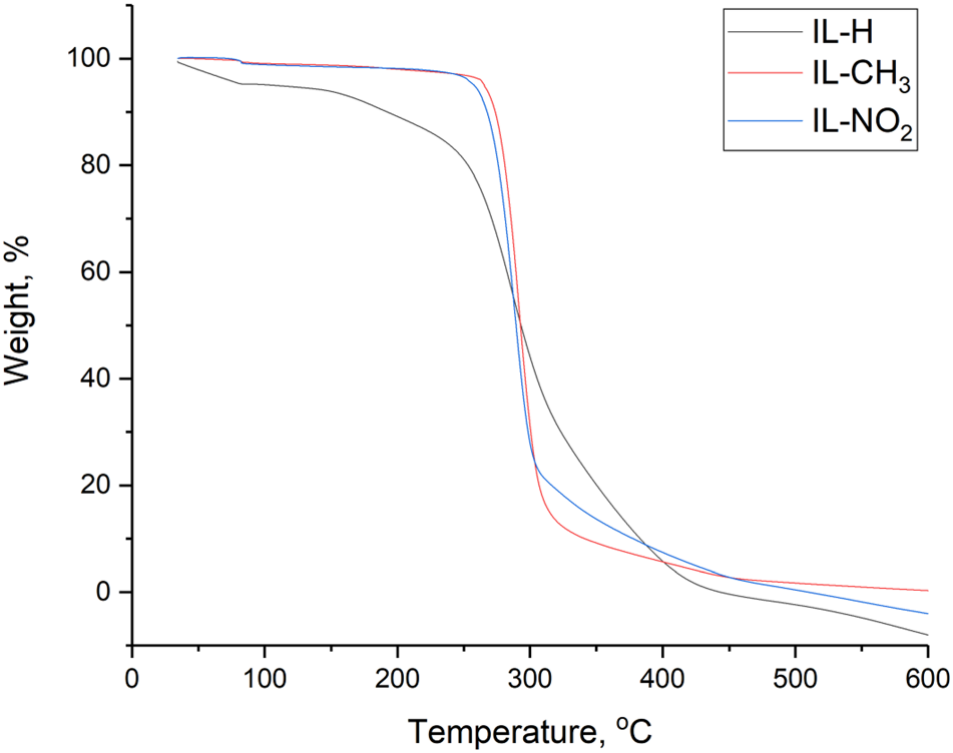
Thermogravimetric analysis (TGA) of IL-H, IL-CH_3_, and IL-NO_2_.

**Figure 8:**
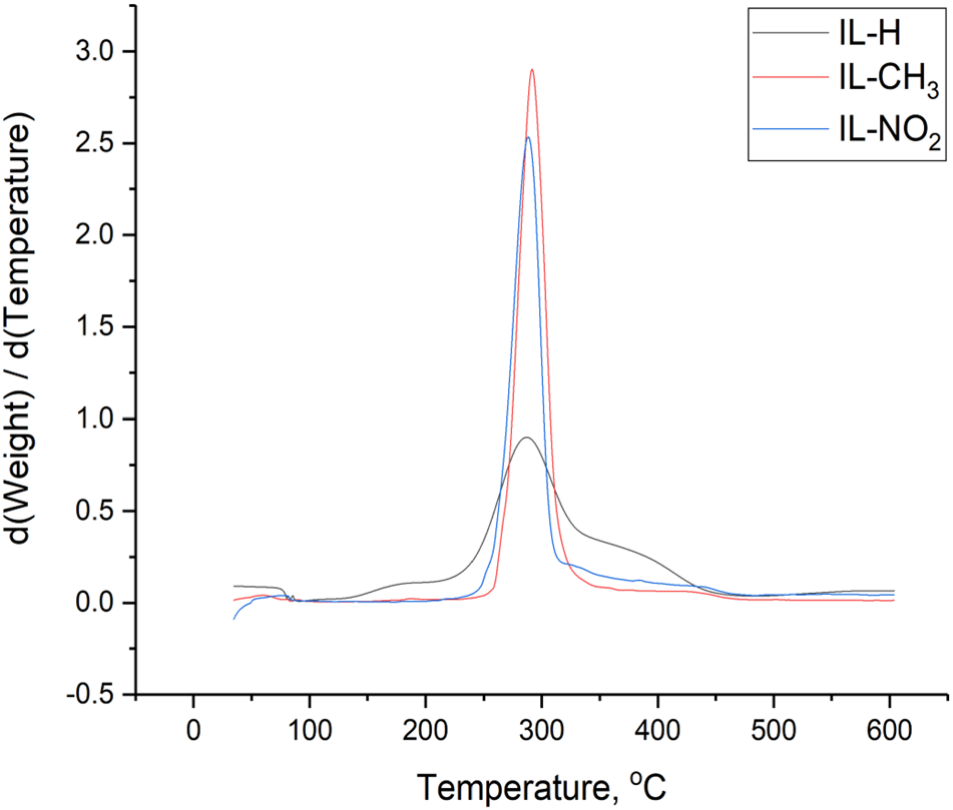
DTG analysis of IL-H, IL-CH_3_, and IL-NO_2_.

TGA and DTG curves indicate that the three prepared ILs started to lose their weights at approximately 280 °C and started the final decomposition around 430 °C as shown in Figs. 7 and 8. Generally, longer the chain length usually results in lower thermal stability; therefore, the first weight loss may be attributed to the decomposition of the attached aliphatic side chain (C_6_H_13_) to the imidazolium ring in the ionic liquid. Regarding to the presented data, IL-H, IL-CH_3_, and IL-NO_2_ possess excellent thermal stability. Such thermally stable ionic liquids may be used to mitigate the flow assurance related issues under different conditions of temperatures and pressures (the use of ionic liquids under high pressure needs more investigation). It can be used upstream inside the oil reservoir or in the pipeline to prevent the deposition of asphaltene, the formation of scale, scavenge H2S, and many other applications after investigation^46^. Moreover, ionic liquids can be efficient as high-temperature lubricants, solvents for high-temperature organic reactions, heat-transfer fluids, and for thermal energy storage.

### Quantum chemical parameters

**Figure 9:**
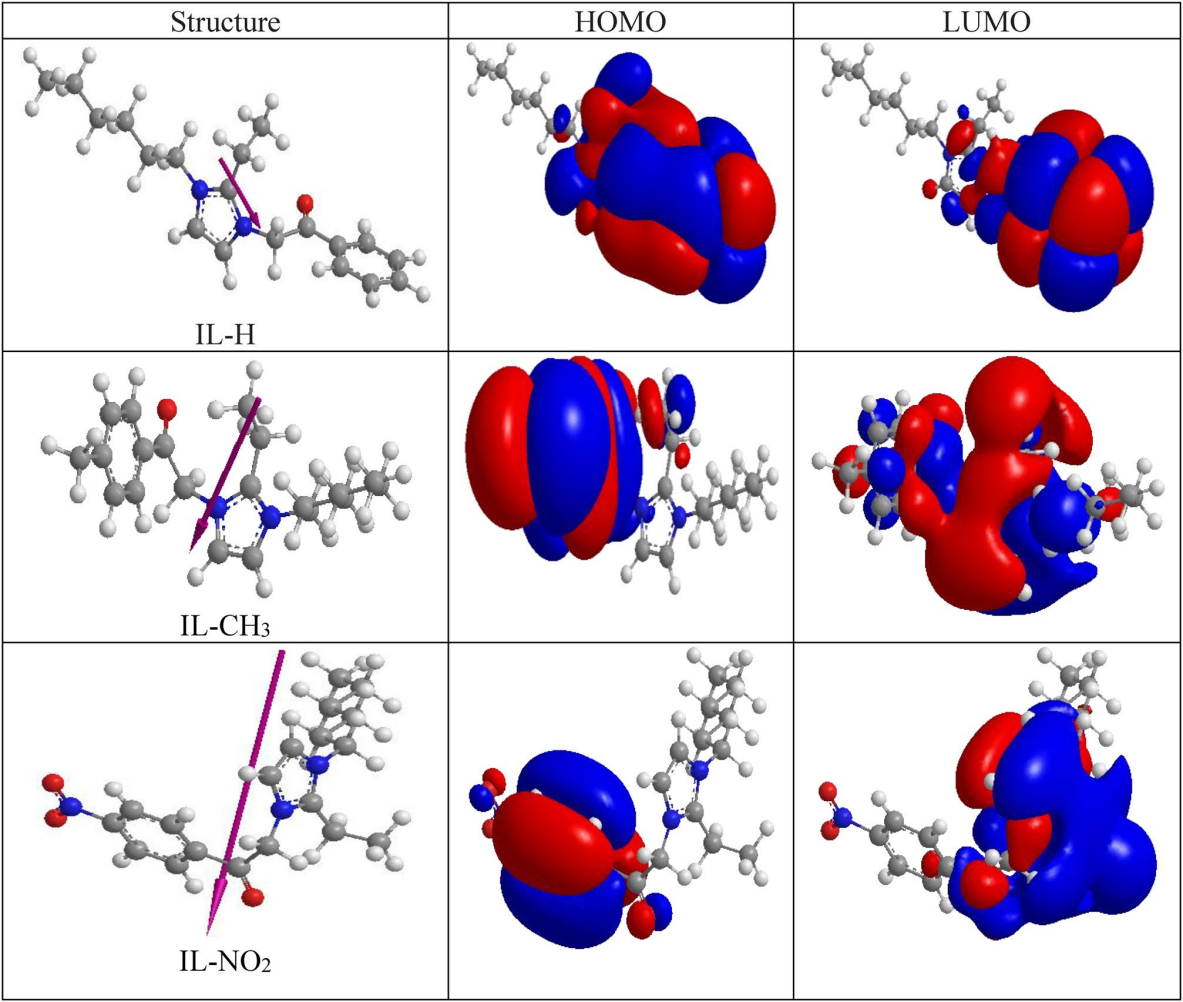
HOMO and LUMO for the prepared ILs.

Molecular orbital calculations can be used to study the electronic structure of materials. Gaussian 09 trial package (HF with basis set 6-31G) was used conduct these calculations for the prepared ILs. Figure 9 shows the optimimized structures, HOMO, and LUMO of IL-H, IL-NO_2_, and IL-CH_3_. The main locations of HOMO and LUMO are concentrated on the imidazolium ring and the aryl chain in the examined molecules. However, the similarities in the molecular structures between the prepared ILs, there are differences in the computed parameters.

The values of different quantum parameters such as the energy of highest occupied molecular orbital (EHOMO), the energy of lowest unoccupied molecular orbital (ELUMO), the gap energy between them (ΔE), ionization potential (I), electron affinity (A), dipole moment (µ), hardness (ɳ), and softness (σ) listed in Table 6. The calculated data showed that the IL-CH_3_ is the most reactive compound because it has the lowest energy gap (ΔE). IL with lower energy gap expresses higher dispersion efficiency of asphaltene, because the required energy to provide electron will be low. In addition, IL-CH_3_ has the highest value of E_HOMO_ (− 4.53 eV), which related to the higher ability of the IL to donate electrons and bond with asphaltene molecules with low consumed energy. Softness is another parameter that can be used to compare the efficiency of the synthesized ILs, where soft molecules (has higher value of softness) are more reactive. In contrast, compounds that have higher hardness values have less reactivity. The values of dipole moment (μ) related to the synthesized compounds are 3.0283, 12.146, and 5.8592 for IL-H, IL-NO_2_, and IL-CH_3_, respectively. However, dipole moment gives a proper investigation of the polarity of a molecule, there is no trend in the resultant values of dipole moment of the prepared dispersants comparing to the other quantum parameters such as ΔE, electron affinity, softness, and hardness^47^.

**Table 6:** The quantum parameters values of the synthesized compounds.

ILs	E_HOMO_, eV	E_LUMO_, eV	ΔE, eV	µ, Debye	A, eV	I, eV	Elect. Negativity, eV mol^−1^	Hardness (ɳ), eV mol^−1^	Softness (σ), eV
IL-H	− 4.75	− 0.369	4.381	3.0283	4.75	0.369	2.5595	2.1905	0.4565
IL-NO2	− 6.826	− 1.265	5.561	12.146	6.826	1.265	4.0455	2.7805	0.3596
IL-CH3	− 4.536	− 0.396	4.14	5.8592	0.396	4.536	2.466	2.07	0.4830

### Efficiency of the prepared dispersants

The prepared ILs were investigated to overcome the asphaltene related issues especially the aggregation during production processes via delaying the precipitation asphaltene onset precipitation (AOP). The AOP before and after treatment was detected using the viscosity measurements as a direct method. It is well-known that any efficient dispersant should have functional groups in order to attach to the other functional groups in the asphaltene molecules via any possible interactions. This process can either dissolve or remove the aggregates^19^. The amount of the asphaltene dispersant should be taken into account, as the dispersion process depends totally on the dispersant to asphaltene ratio. Therefore, different concentrations of the prepared ILs were investigated to detect the optimum amount.

#### Detection of the AOP

**Figure 10:**
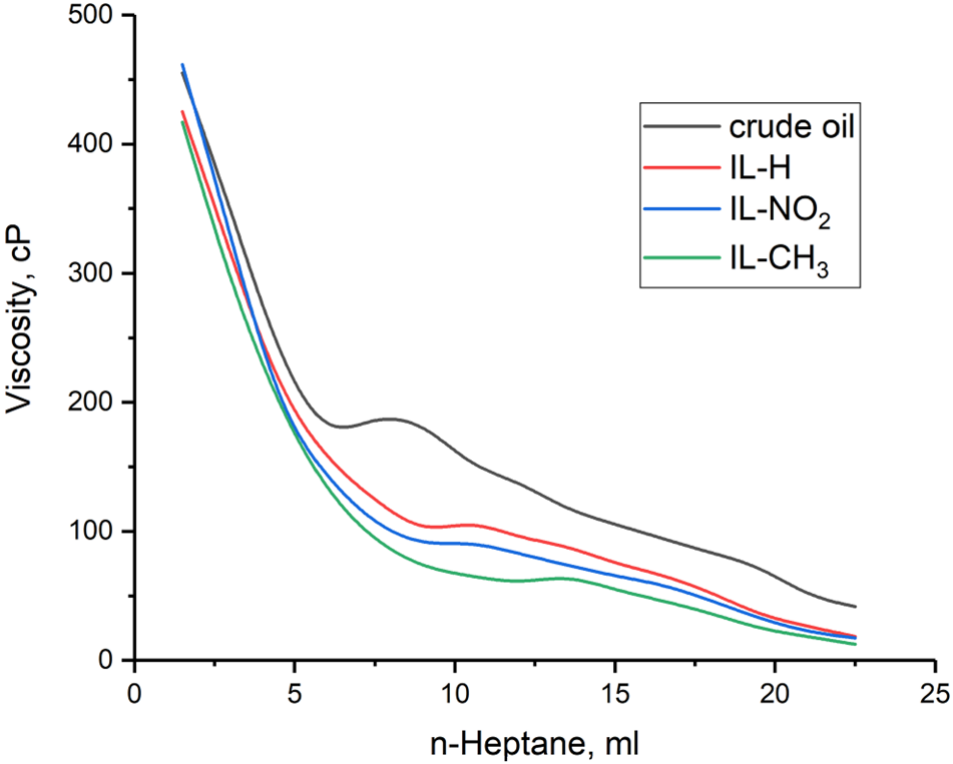
Asphaltene onset precipitation (AOP) before and after using 1500 ppm of IL-H, IL-NO_2_, and IL-CH_3_.

The AOP can be detected using measuring the viscosity of the titrated oil sample with n-heptane. The presence of enough amount of resin could stabilize asphaltene as a suspension in the colloidal system, and this is because of polar-polar interactions between resin and asphaltene^48^. Therefore, any decrease in resin amount effect on asphaltene precipitation, also any increase in aliphatic solvent has the same effect. The effect of IL-H, IL-CH3, and IL-NO_2_ on the AOP process is shown in Fig. 10. It is obvious that there is a general decrease in the viscosity of the crude oil mixture while the amount of n-heptane increases until it reaches a critical point, at which the viscosity dramatically increases. This point is known as the asphaltene onset precipitation (AOP). According to Fig. 10, 1500 ppm of IL-H, IL-NO_2_, and IL-CH_3_ delayed the AOP from 7.5 to 10.5, 11, and 13.5 ml *n*-heptane, respectively. The structure of the prepared ILs was designed to be similar to the resin structure and to have a similar effect on asphaltene molecules, therefore, increasing the amount of the prepared ionic liquids, increasing their efficiency as asphaltene inhibitors as shown in Table 7.

**Table 7:** Effect of different concentrations of the prepared ILs on the AOP.

Concentration	AOP (n-heptane, ml)		
	IL-H	IL-NO_2_	IL-CH_3_
1000, ppm	8	10	10
1500, ppm	10.5	11	13.5
2000, ppm	12	11.5	14

#### Colloidal instability index

It is reported by Carbognani and Espidel that the instability of any oil system is not accompanied by the asphaltene content itself, but it is related to the instability of asphaltene in the media^49^. Therefore, SARA analysis is used to investigate the stability of asphaltene in the crude oil by calculating the CII. According to Eq. (1) and Table 8, the CII of the crude oil sample is equal to 1.8. This means that the asphaltene in the crude oil sample is unstable and has a great opportunity to precipitate. Asphaltene in the crude oil is stable for CII ≤ 0.7, and unstable for CII ≥ 0.9, while the range of 0.7 < CII > 0.9 means that asphaltene is metastable and the stability is not clear^50^. It is obvious that the crude oil sample contains a high amount of asphaltene, which is one of the most important reasons for the crude oil sample’s high viscosity. The data in Table 8 showed that the resin content of the untreated crude oil is slightly low, which is a major factor in increasing the colloidal instability index value. From the colloidal point of view, resin is the main fraction that can stack with and surround the asphaltene molecules to prevent precipitation. In our case, the content of resin is not enough to surround all the asphaltene molecules and disperse them in the medium. The instability of crude oil also depends on saturate content, where a high amount of saturates means a low content of both aromatics and resins. This result in a high value of CII, and destabilize the asphaltene molecules in the oil. The effect of the added ILs on the CII of the crude oil is shown in Table 8, where the asphaltene content is decreased to 13.8 after using IL-CH_3_. Moreover, the amount of resin increased significantly. It is obvious that the colloidal instability index decreased from 0.91 to 0.69, which is a stable value for the oil system. Attachment of an aryl group to the imidazolium IL increases the homogeneity and the attraction force between the IL dispersant and the asphaltene molecules. Moreover, according to Atta et al., the presence of an alkyl chain attached to the IL has a positive effect on the asphaltene dispersion process^51^.

**Table 8:** SARA analysis and CII of both crude and treated oil with IL-CH_3_.

Oil fraction	Method	Crude oil	Treated oil
Saturates	ASTM D2007	32.53	27.1
Aromatics		32.63	35.3
Resin		19.7	23.8
Asphaltene	IP 143	15.14	13.8
CII	Equation (1)	0.91	0.69

#### Effect of ILs on the compositional analysis

Chromatographic analysis of crude oil before and after using IL-CH_3_ at its optimum concentration is shown in supplementary material Fig. S1, while the molar percent of each component is tabulated in Table 9. It is clear that there is a large fraction (up to 57 mol. %) with heavy components starting from pentaicosanes (C_25_) to tetracontanes plus (C_40_^+^). This is due to the high percentage of asphaltene (15.14 wt. %) in the crude oil. GC analysis shows a change in the oil composition after treatment with IL-CH_3_, where the content of light hydrocarbons, *n*-dodecanes (C_12_) to *n*-nonadecanes (C_21_), increased to a large extent (up to 2.2 mol.%), while the heavy hydrocarbons, icosans (C_25_) to hexatricontanes (C_40_^+^) decreased. Moreover, the in-between fractions, icosanes (C_20_) to tetraicosanes (C_24_) showed consistency in their content largely. The changes in oil composition led to a decrease in molecular weight from 360 to 326. All these changes were affected by the dispersion power of asphaltene aggregates after using IL-CH_3_.

**Table 9:** Mole percentage of the crude oil before and after asphaltene dispersion using IL-CH_3_.

Component		Crude oil	Treated oil
		Mole %	Mole %
Dodecanes	C_12_	0.000	0.11814
Tridecanes	C_13_	0.47623	0.69896
Tetradecanes	C_14_	1.14295	2.75650
Pentadecanes	C_15_	3.04786	4.82387
Hexadecanes	C_16_	2.95262	5.02077
Heptadecanes	C_17_	5.52425	8.95861
Octadecanes	C_18_	6.38146	9.05706
Nonadecanes	C_19_	3.33360	5.02077
Icosanes	C_20_	3.09548	4.62697
Eneicosanes	C_21_	2.95262	2.93277
Dodeicosanes	C_22_	5.12109	5.31415
Tricosanes	C_23_	4.46931	4.52462
Tetraicosanes	C_24_	4.28309	4.32773
Petaicosanes	C_25_	5.44805	4.37102
Hexaicosanes	C_26_	3.91996	4.14459
Heptaicosanes	C_27_	5.38138	4.04910
Octaicosanes	C_28_	4.95278	3.93027
Nonaicosanes	C_29_	4.74324	3.91058
Tricontanes	C_30_	4.42892	3.65687
Entricontanes	C_31_	4.09556	3.40092
Dodetricontanes	C_32_	3.80030	3.00261
Tritricontanes	C_33_	3.68601	2.80798
Tetratricontanes	C_34_	3.41932	2.30365
Pentatricontanes	C_35_	3.26693	1.87048
Hexatricontanes	C_36_	3.09548	1.47670
Hepatricontanes	C_37_	2.67640	1.26995
Octatricontanes	C_38_	2.04778	0.83680
Nonatricontanes	C_39_	1.59060	0.44301
Tetracontanes	C_40_	0.66672	0.34456
Total		100.000	100.000
Molecular weight		360.997	326.101

### Dispersion mechanism

The IL acts as an asphaltene dispersant or inhibitor regarding to the delivered crude oil properties and the related properties of either the IL’s polar head or the hydrocarbon tail. It is well known that the asphaltene molecule demonstrates a strong H-bond acceptor and a weak donor. Moreover, the lone pair of electrons of the heteroatoms (N, O, and S) that are attached to the asphaltene molecules may play a role in the charge transfer^52^. The competing interactions between the anion and the cation of the ionic liquid and the asphaltenes aggregates, which are of varying strength, may lead to a new system of hydrogen-bonded formation or charge-transfer complexes with high stabilization energies. These energies are known as “the energy of interaction” between the IL dispersant and the asphaltene molecule^40^.

**Figure 11:**
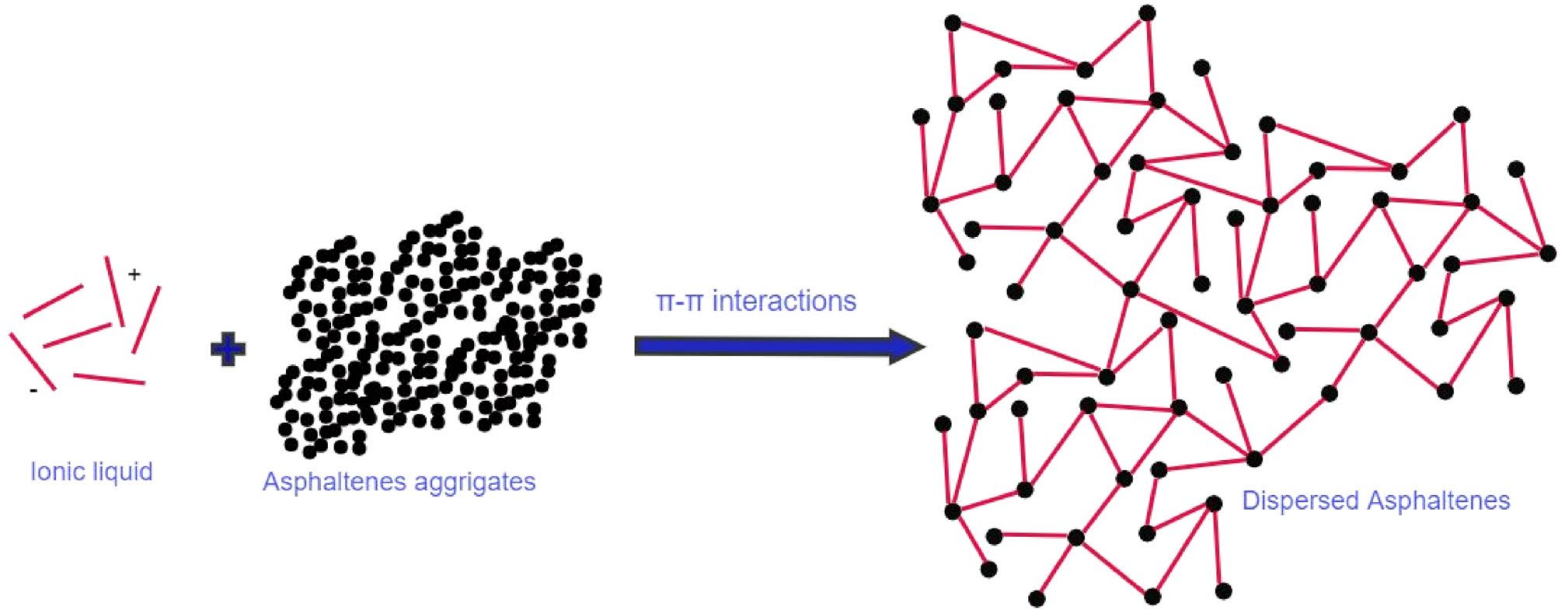
Suggested mechanism for the dispersion of asphaltene aggregates using ILs.

The selected ILs for this study as asphaltene dispersants were influenced regarding to different parameters including their high solubility in the oil medium and the intricacy of their interactions. ILs displayed high dispersion performance of asphaltene molecules possibly due to the imidazolium ring and the benzene ring (rich in π electrons) in the ionic liquid can make the π–π interaction with the aromatic rings in the asphaltene molecules. Therefore, they could disturb and disperse the aggregated asphaltenes through the π–π interactions as shown in Fig. 11. This is attributed to the charge transfers from ILs to asphaltene molecules because of their specific donor–acceptor properties making the asphaltenes more stable and ultimately preventing further asphaltenes aggregation or precipitation. The chloride anion (non-bonding electron) in the crude oil environment acts as a source of electron donors, which can attach to the asphaltenes π–electrons^53^. Therefore, the difference in charge density between both the anion and the cation support the anion [Cl]^−^, which is suited to conduct both the H-bonding interactions or/and electron donor/acceptor interactions with asphaltenes and inhibit their aggregations. In addition, asphaltene molecules contain a high degree of electron density in their π–electron system, which make them more sensitive to nucleophilic attack (electron donating groups as in IL-CH_3_). Moreover, the presence of the prepared ILs in keto-enol form could enhance the dispersion of asphaltene aggregates due to the formation of hydrogen bonds with the asphaltene molecules. Therefore, IL-CH_3_ is more likely to interact with asphaltene through nucleophilic interactions, leading to better solubility and dispersion of the aggregated asphaltene molecules. It is important to conduct more research on the mechanism of interactions of asphaltene and its dispersant, because these interactions are complicated and depends on a wide range of variables^54^.

## Conclusions

Colloidal instability index is used to recognize the asphaltene precipitation potential of crude oil system. Asphaltene molecules are mainly stabilized in the crude oil medium by the resin molecules. Most of the asphaltene dispersants are designed to mimic the structure of resin. Therefore, three ionic liquids containing different aryl chains were synthesized and were investigated as asphaltene dispersants using the viscometric method. Based on the obtained results, it can be concluded that:

1. The prepared materials were characterized using different spectroscopic techniques such as FT-IR and NMR. The thermal stability of the prepared ILs was studied using TGA.
2. The experimental data showed that the ILs have a significant impact on the dispersion of the asphaltene aggregates. This is due to the presence of the attached aryl chain to the imidazolium ionic liquid, which can mimic the resin structure and disperse asphaltene.
3. IL-CH_3_ is the most effective ionic liquid due to the presence of alkyl chain in its structure. The colloidal instability index of the crude oil medium was changed from unstable to stable crude after the addition of the optimum concentration of IL-CH_3_.
4. Quantum chemical parameters such as E_HOMO_, E_LUMO_, energy gap, and dipole moment were calculated. The results showed that the presence of alkyl chain in the dispersant structure increases the reactivity of the ionic liquid. However IL-NO_2_ is the most polar compound and has the highest dipole moment, IL-CH_3_ was the preferred dispersant.
5. These results were in a great compliance with the experimental data.

### Supplementary Information

Supplementary Figure S1.

## Data Availability

The data that support the findings in the present study are available from the corresponding author upon request.
