# Supplementary material for: Imidazolium-based ionic liquids as dispersants to improve the stability of asphaltene in Egyptian heavy crude oil
Source: Sci Rep. 2023 Oct 11;13:17158. doi: 10.1038/s41598-023-44237-w (PMC10567904; doi:10.1038/s41598-023-44237-w)
Supplement: Supplementary file 1 — Supplementary Figure S1. [file 41598_2023_44237_MOESM1_ESM.docx]

**Imidazlium-Based Ionic Liquids as Dispersants to Improve the Stability of Asphaltene in Egyptian Heavy Crude Oil**

Alaa Ghanem ^1,2^*, Maher I. Nessim^3^, N.A. Khalil^3^, Raghda A. El-Nagar ^3,^*

^1^PVT lab, Production Department, Egyptian Petroleum Research Institute, Nasr City, Cairo, 11727, Egypt.

^2^ PVT Services Center, Egyptian Petroleum Research Institute, Nasr City, Cairo, 11727, Egypt.

^3^ Petroleum Testing Lab, Analysis & Evaluation Department, Egyptian Petroleum Research Institute, Nasr City, Cairo, 11727, Egypt.

Corresponding Email: [alaa_ghanem2001@yahoo.com](mailto:alaa_ghanem2001@yahoo.com) & [alaa_ghanem2001@epri.sci.eg](mailto:alaa_ghanem2001@epri.sci.eg)


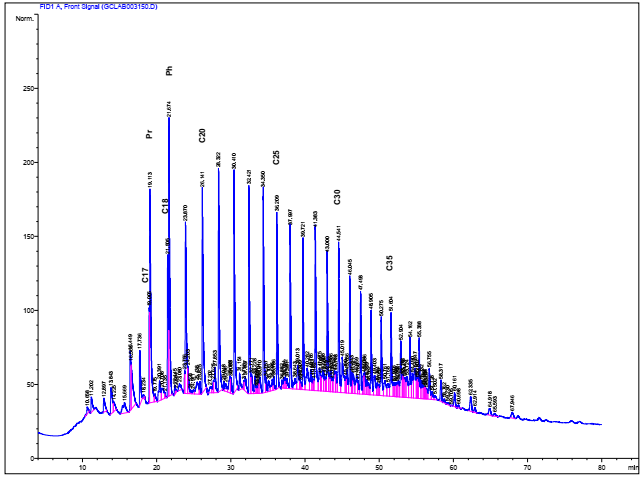


(a)


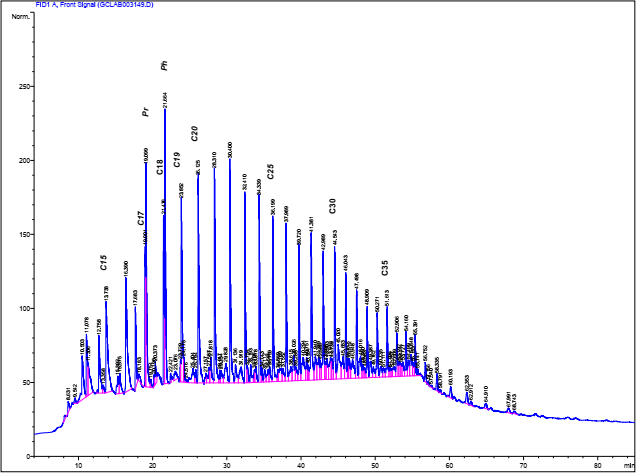


(b)

Figure S1 Gas chromatographic analysis of crude oil (a) before and (b) after asphaltene dispersion
